# How to schedule night shift work in order to reduce health and safety risks

**DOI:** 10.5271/sjweh.3920

**Published:** 2020-10-30

**Authors:** Anne Helene Garde, Luise Begtrup, Bjørn Bjorvatn, Jens Peter Bonde, Johnni Hansen, Åse Marie Hansen, Mikko Härmä, Marie Aarrebo Jensen, Göran Kecklund, Henrik A Kolstad, Ann Dyreborg Larsen, Jenny Anne Lie, Claudia RC Moreno, Kirsten Nabe-Nielsen, Mikael Sallinen

**Affiliations:** 1 The National Research Centre for the Working Environment, Copenhagen, Denmark; 2 Department of Public Health, University of Copenhagen, Copenhagen, Denmark; 3 Department of Occupational and Environmental Medicine, Bispebjerg and Frederiksberg Hospitals, Copenhagen, Denmark; 4 Department of Global Public Health and Primary Care, University of Bergen, Bergen, Norway; 5 Norwegian Competence Center for Sleep Disorders, Haukeland University Hospital, Bergen, Norway; 6 Danish Cancer Society Research Center, Copenhagen, Denmark; 7 Finnish Institute of Occupation Health, Helsinki, Finland; 8 Stress Research Institute, Department of Psychology, Stockholm University, Stockholm, Sweden; 9 Department of Occupational Medicine, Danish Ramazzini Centre, Aarhus University Hospital, Aarhus, Denmark; 10 National Institute of Occupational Health, Oslo, Norway; 11 School of Public Health, University of São Paulo, Sao Paulo, Brazil

**Keywords:** cancer, cardio-metabolic disease, circadian disruption, injury, night work, pregnancy, shift work schedule, shift worker, sleepiness, sleep duration, sleep quality

## Abstract

**Objectives:**

This discussion paper aims to provide scientifically based recommendations on night shift schedules, including consecutive shifts, shift intervals and duration of shifts, which may reduce health and safety risks. Short-term physiological effects in terms of circadian disruption, inadequate sleep duration and quality, and fatigue were considered as possible links between night shift work and selected health and safety risks, namely, cancer, cardio-metabolic disease, injuries, and pregnancy-related outcomes.

**Method:**

In early 2020, 15 experienced shift work researchers participated in a workshop where they identified relevant scientific literature within their main research area.

**Results:**

Knowledge gaps and possible recommendations were discussed based on the current evidence. The consensus was that schedules which reduce circadian disruption may reduce cancer risk, particularly for breast cancer, and schedules that optimize sleep and reduce fatigue may reduce the occurrence of injuries. This is generally achieved with fewer consecutive night shifts, sufficient shift intervals, and shorter night shift duration.

**Conclusions:**

Based on the limited, existing literature, we recommend that in order to reduce the risk of injuries and possibly breast cancer, night shift schedules have: (i) ≤3 consecutive night shifts; (ii) shift intervals of ≥11 hours; and (iii) ≤9 hours shift duration. In special cases – eg, oil rigs and other isolated workplaces with better possibilities to adapt to daytime sleep – additional or other recommendations may apply. Finally, to reduce risk of miscarriage, pregnant women should not work more than one night shift in a week.

In 2007 and again in 2019, the International Agency for Research on Cancer (IARC) classified night shift work as probably carcinogenic to humans (group 2A) based on limited evidence of cancer in humans, sufficient evidence of cancer in experimental animals, and strong mechanistic evidence in experimental animals (1). Night shift work was defined as work of ≥3 hours between 23:00–06:00 hours and may be organized in many ways including 2- or 3-shift work, irregular schedules, and permanent night shift work (2, 3). The 2007 IARC evaluation prompted a workshop in Denmark resulting in recommendations for the prevention of the effects of night shift work on risk of breast cancer based upon the available epidemiological, experimental and mechanistic evidence at that time (4). Since then, numerous epidemiological and experimental studies on different types of cancer have been published. Furthermore, the risk of other adverse health outcomes, such as cardiovascular disease, diabetes, injuries and pregnancy-related outcomes, have also been associated with night shift work. The increasing amount of studies on shift work, health and safety has prompted requests from policy-makers, employers and employees for scientifically based recommendations on specific ways to schedule night shift work in order to reduce health and safety risks, which extend previous recommendations on breast cancer to other outcomes.

This paper aims to provide scientifically based recommendations on night shift schedules that reduce health and safety risks. As outlined in figure 1, *night shift schedules* included night shift intensity (number of night shifts per unit time), consecutive night shifts (number of night shifts in a row), permanent night shift work (primarily or only night shifts), shift intervals (time between shifts), direction of rotation (typically forward, eg, D → E → N, or backward rotation, eg, N → E → D) and shift duration (number of hours in a shift). The a priori selected *health and safety outcomes* were cancer, cardio-metabolic disease, injuries and pregnancy-related outcomes, such as miscarriage and pregnancy-related hypertension and pre-eclampsia. These outcomes were selected because of severity of the disease/event and prior knowledge of studies expected to be informative. Furthermore, *short term physiological effects* related to circadian disruption, inadequate sleep duration and quality, fatigue and sleepiness were considered as possible mechanisms linking night shift work to health and safety risks and considered for further evidence in the formulation of the given recommendations.

**Figure 1:**
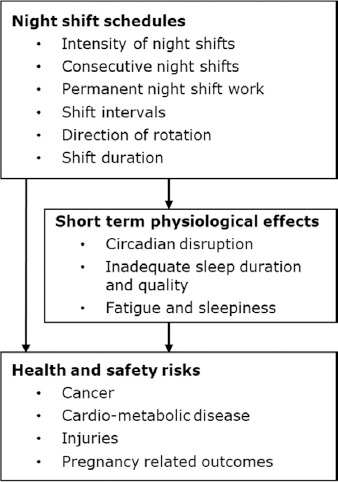
Outline of selected core shift work schedules, potential mechanisms and selected outcomes included in the present work.

## Methods

Working within different fields of night shift work and health and safety research and performing epidemiological, observational or experimental studies, 15 experience shift work researchers participated in a 3-day workshop held in January 2020 in Denmark. Prior to the workshop, the participants identified the most relevant scientific literature on the associations and possible mechanisms between night shift work and health and safety within their main research area. After the workshop, a supplementary literature search was performed in PubMed. Furthermore, studies included in recent systematic reviews were checked for relevant information. Cohort and case–control studies and meta-analysis that assessed two or more doses or categories of a night shift schedule were included. Cross-sectional studies and studies that solely compared night shift work with day or non-night work, eg, ever/never night, were excluded. The recommendations were based on the literature on night shift schedules and the a priori selected outcomes. Evidence on short-term physiological effects was used to substantiate the recommendations.

## Results

### Short-term physiological effects of night shift work on health and safety risks

*Circadian disruption*. Circadian rhythms in physiological functions are pivotal for survival (5). They are primarily synchronized to the light–dark cycle by light exposure through the eyes, which excites the intrinsically photosensitive retinal ganglion cells (ipRGC). The ipRGC are connected to the suprachiasmatic nucleus (SCN) located in the hypothalamus (6). Virtually all cells in the body have molecular clocks that are normally synchronized by the master clock in the SCN. Projections from the SCN innervate the sympathetic nervous system and other structures such as the pineal gland, which regulates downstream peripheral oscillators via humoral, endocrine, and neural signals, resulting in a coherent time organization of bodily processes for optimal performance (7). Melatonin is a hormone mainly produced in the pineal gland under direct control of the circadian timing system. Thus, melatonin production is controlled by the light–dark cycle exposure, and its plasma concentration signalizes this to virtually all organs and tissues. Therefore, melatonin is essential to maintain the internal circadian synchronization and regulate the sleep–wake cycle.

In this paper, the term “circadian disruption” is used in a broad sense to cover the changes in the circadian rhythm such as amplitude, duration, and timing of biological rhythms and objective or subjective proxies of changed circadian rhythm (8). Circadian disruption may, depending on intensity and duration, be caused by a number of factors: light-at-night, altered sleep–wake cycle (disturbed sleep), and other behaviors that alter the peripheral clocks (9).

Night shift workers are exposed to light-at-night, which has earlier been proposed as one of the exposures linked with breast cancer through suppression of melatonin (10). Light-at-night is associated with lower levels of melatonin in both experimental (11–13) and observational studies of night shift workers compared with day workers (14, 15). Animal studies show that alterations in the light–dark schedule suppress melatonin (3). Furthermore, some prospective cohort studies indicate that women with higher levels of morning urinary melatonin metabolites have a modestly lower risk of breast cancer (16, 17).

For optimal biological efficiency, key circadian rhythms must maintain a certain phase relation to one another and follow an internal order (7). Because different organ tissues, biological systems and cells change their rhythms with different speed, desynchronization between internal circadian rhythms may develop as a consequence of night shift work (18). Experimental evidence shows that the internal desynchronization leads to an accelerated growth of human breast tumor xenografts in mice (19). The degree of desynchronization may depend on type of night shift schedules, eg, suppression of melatonin and changes in cortisol rhythms were influenced by the number of increasing number of consecutive night shifts among Danish male police officers (20).

*Sleep duration and quality*. Night shift workers normally sleep during the day, which is associated with short sleep duration, insomnia symptoms such as premature awakening and non-restorative sleep, and a reduction in stage 2 and rapid eye movement (REM) sleep compared with other shifts and days off (21–23). It is likely that components of the shift schedule, for example number of days off, intensity of night shift work, and frequency of quick returns and early morning work, contribute to chronic short sleep duration and insomnia (24). If the night shift worker suffers from shift work disorder, defined as having shift work-related sleep problems and/or excessive sleepiness, one would assume that the sleep disturbances are chronic. The prevalence of shift work disorder is higher among night shift workers than shift workers who alternate between day and evening work, and shift work disorder is also positively correlated with frequency of night shifts in the shift schedule (25, 26).

Chronic short sleep duration (≤6 hours per day), particularly when associated with insomnia complaints, has been associated with cardiovascular disease and type-2 diabetes (27, 28) and could be a mechanism linking night shift work with these adverse health outcomes. It has also been hypothesized that short sleep duration may increase cancer risk and that long sleep may reduce breast cancer, but findings are inconsistent (29). However, to the best of our knowledge, no prospective study has evaluated whether shift work disorder or chronic short sleep duration among night shift workers increase the risk of developing cardio-metabolic diseases and cancer.

*Fatigue and sleepiness*. Fatigue and sleepiness, particularly related to sleep restriction, have been suggested as plausible mechanisms linking night shift work and injury through impaired performance and alertness (30). Sleepiness, defined as increased sleep pressure, has been shown to rise while working during the night and may depend on the night shift schedule. The most consistent result is that sleepiness is most profound on the first night shift in both experimental and observational studies (31–38). Also, slowly backward-rotating shift systems (which have several consecutive shifts and sometimes short shift intervals) have been found to be associated more strongly with sleepiness on night shifts than fast forward-rotating systems (39). Furthermore, there are indications that alertness and performance are impaired on 12-hour night shifts compared with 8-hour shifts (40, 41), although later studies did not find such an effect on sleep or sleepiness (39).

Taken together, night shift work has several short-term physiological effects: circadian disruption is introduced, levels of melatonin are modestly suppressed, circadian rhythms are desynchronized, sleep duration is reduced, and sleepiness is increased. The short-term physiological effects appear to depend on how the night shifts are scheduled (20, 25, 26, 31–38). The short-term physiological effects are suggested to link night shift work to acute safety risks and possibly long-term health effects, although studies specifically addressing whether these acute effects serve as mediators of long-term health and safety risks are lacking.

### Scheduling of night shift work and risk of cancer, cardio-metabolic disease and injuries

Studies of night shift intensity, consecutive night shifts, shift intervals, and duration of shift in relation to risk of cancer, cardio-metabolic disease and injuries are summarized in tables 1–3. In table 4, results from studies on pregnancy-related outcomes are presented.

**Table 1:** Studies on intensity of night shifts and number of consecutive night shifts and risk of cancer, cardio-metabolic disease and injuries. [NA=not available; CI=confidence interval.]

Study	Study design	Outcome	Exposure	Cases	Risk	95% CI
Davis, Mirick, & Stevens, 2001 (43)	Case–control study (1993–1995)	Breast cancer	Night work hours/week (avg. of 10 year period)			
			0	713	1	Reference
			<1.2	11	1.3	0.5–3.1
			1.2–2.7	13	1.4	0.6–3.2
			2.7–5.7	13	1.5	0.6–3.6
			≥5.7	17	2.3	1.0–5.3
Lie et al, 2011 (49) control study of nurses	Nested case– (1990–2007)	Breast cancer	Never night work	102	1	Reference
			<5 years night shift with # of consecutive shifts:			
			≥3	194	1.1	0.8–1.6
			≥4	160	1.2	0.8–1.6
			≥5	137	1.2	0.8–1.7
			≥6	119	1.2	0.8–1.7
			≥7	109	1.1	0.8–1.6
			≥5 years night shift with # of consecutive shifts:			
			≥3	278	1.1	0.8–1.5
			≥4	131	1.4	0.9–1.9
			≥5	74	1.6	1.0–2.4
			≥6	64	1.8	1.1–2.8
			≥7	58	1.7	1.1–2.8
			Years with ≥3 night shifts/month			
			Never night work	102	1	Reference
			Never ≥3 night shifts/month	28	1.4	0.8–2.6
			1–14	390	1.2	0.9–1.6
			15–29	152	1.2	0.9–1.7
			≥30	27	0.8	0.5–1.4
Hansen & Lassen, 2012 (45)	Nested case–control study of military employees (1990–2003)	Breast cancer	Consecutive night shifts/week			
			0	82	1	Reference
			1–2	15	1.0	0.5–1.9
			≥3 for 1–5.9 years	9	1.1	0.5–2.3
			≥3 for 6–14.9 years	11	2.1	1.0–4.8
			≥3 for ≥15 years	9	2.5	1.0–6.6
Fischer et al 2017 (51)	Meta-analysis	Occupational injuries	Consecutive night shifts			
			1^st^	NA	1	Reference
			2^nd^	NA	1.05	0.92–1.21
			3^rd^	NA	1.16	0.96–1.40
			4^th^	NA	1.36	1.14–1.62
Cordina-Duverger et al, 2018 (46)	Pool of five harmonized case–control studies (2004–2013)	Breast cancer	Premenopausal women			
			Night shifts/week			
			Never	1393	1	Reference
			<1	62	1.31	0.89–1.93
			1–2	108	1.03	0.78–1.36
			≥3	68	1.80	1.20–2.71
			Postmenopausal women			
			Night shifts/week			
			Never	2979	1	Reference
			<1	60	0.73	0.51–1.03
			1–2	146	1.01	0.79–1.29
			≥3	64	0.92	0.65–1.31
Wendeu-Foyet et al, 2018 (50)	Case-control study (2012–2014)	Prostate cancer	Never night work	532	1	Reference
			Permanent night workers			
			Consecutive night shifts			
			<6	95	1.01	0.74–1.39
			≥6	93	1.33	0.95–1.87
			Shift length (hour) for number of consecutive nights			
			≤10 for <6	13	0.54	0.27–1.09
			≤10 for ≥6	18	0.58	0.32–1.07
			>10 for <6	4	0.60	0.16–2.15
			>10 for ≥6	30	2.57	1.31–5.06
			Rotating night workers			
			Consecutive night shifts			
			<6	58	0.77	0.53–1.11
			≥6	26	0.98	0.56–1.74
			Shift length (hour) for number of consecutive nights		
			≤10 for <6	50	0.72	0.49–1.06
			≤10 for ≥6	22	0.93	0.50–1.71
			>10 for <6	8	1.24	0.44–3.55
			>10 for ≥6	4	1.36	0.29–6.26
Vetter al. 2018(44)	Cohort study (2006–2015)	Type 2 diabetes	Current type of night shift work			
			Day work	5173	1	Reference
			Shift work with no/rare nights	730	1.11	1.02–1.22
			Rotating shifts with some nights	461	1.13	1.01–1.22
			Rotating shifts with usual nights	169	1.37	1.13–1.65
			Average lifetime night shifts/month			
			0	804	1	Reference
			<3	52	1.16	0.83–1.58
			3–8	125	1.02	0.82–1.26
			>8	210	1.21	1.02–1.45
Nielsen et al, 2018 (48)	Register-based cohort study of hospital employees in Denmark (2007–2015)	Injuries	Night shifts in a week			
			0	2161	1	Reference
			1	603	1.12	0.98–1.28
			2	352	1.02	0.88–1.17
			3	171	0.95	0.80–1.12
			4	64	1.00	0.78–1.30
			≥5	29	1.08	0.73–1.59
Ferguson et al, 2019 (47)	Cohort study of aluminum manufacturing workers (2003–2013)	Hypertension	Average % night shifts/month in the past year			
			0	26	1	Reference
			>0–5	34	2.47	1.12–5.44
			>5–50	98	2.40	1.04–5.55
			>50–95	42	3.21	1.32–7.80
			>95–100	15	3.71	1.24–11.09
Härmä et al 2020 (52)	Register-based case-crossover study of hospital employees in Finland (2003–2015)	Injuries	Night shifts in a week			
			0	18 837	1.03	0.97–1.10
			1	830	1.12	1.01–1.23
			2	920	0.94	0.85–1.03
			3	409	0.85	0.74–0.98
			4	209	0.87	0.70–1.08
			>5	125	0.81	0.59–1.12

**Table 2:** Studies on shift intervals and risk of injuries. ^a^ [CI=confidence interval.]

Study	Study design	Outcome	Exposure	Cases	Risk	95% CI
Nielsen et al, 2019 (61)	Register-based cohort study of hospital employees in Denmark (2008–2015)	Injuries	Time between shifts (hours)			
			1–2	17	1.52	0.94–2.45
			3–5	19	2.24	1.42–3.53
			6–8	116	1.36	1.13–1.64
			9–11	107	1.32	1.09–1.60
			12–14	232	1.24	1.09–1.41
			15–17	4597	1	Reference
			Quick return (6–11 hours) before a:			
			Day shift	327	1	Reference
			Evening shift	38	1.32	0.94–1.85
			Night shift	76	0.91	0.70–1.17
Vedaa et al, 2020 (62)	Longitudinal cohort study of nurses (2016–2018)	Harmed oneself	1–34 quick returns/year at baseline and follow–up	120	1	Reference
			Increase from 1–34 to >35 quick returns/year	148	2.58	0.71–9.34
			>35 quick returns/year at baseline and follow–up	64	1	Reference
			Decrease from >35 to 1–34 quick returns/year	271	0.35	0.16-0.73
		Harmed patients	1–34 quick returns/year at baseline and follow–up	120	1	Reference
			Increase from 1–34 to >35 quick returns/year	148	8.49	2.79–25.87
			>35 quick returns/year at baseline and follow–up	64	1	Reference
			Decrease from >35 to 1–34 quick returns/year	271	0.27	0.12-0.59
Härmä et al 2020 (52)	Register-based case-crossover study of hospital employees in Finland (2003–2015)	Injuries	Number of quick returns in the preceding 7 days			
			0	18 636	0.97	0.86–1.09
			1	36	0.84	0.53–1.35
			2	18	1.40	0.68–2.89
			3	10	1.13	0.45–2.76
			>4	10	1.00	0.41–2.46

a No studies on cancer and cardio-metabolic diseases were found.

**Table 3:** Studies on daily duration of shifts and risk of cancer and injuries.^a^ [NA=not available; CI=confidence interval.]

Study	Study design	Outcome	Exposure	Cases	Risk	95% CI
Fischer et al, 2017 (51)	Review and meta-analysis	Occupational injuries	Shift length (hours)			
			8	NA	1	Reference
			9	NA	1.06	0.69–1.63
			10	NA	1.54	1.30–1.83
			11	NA	1.51	1.30–1.74
			12	NA	1.77	1.50–2.07
			>12	NA	2.73	2.02–3.69
Cordina-Duverger et al, 2018 (46)	Pool of five harmonized case-control studies (2004–2013)	Breast cancer	Pre-menopausal women			
			Length of night shift (hours)			
			Never night work	1669	1	Reference
			<8	37	1.03	0.65–1.64
			8–9	111	1.20	0.91–1.60
			≥10	167	1.36	1.07–1.74
			Post-menopausal women			
			Length of night shift (hours)			
			Never night work	3652	1	Reference
			<8	47	1.09	0.73–1.65
			8–9	213	1.12	0.92–1.36
			≥10	177	0.96	0.78–1.19
Wendeu-Foyet et al, 2018 (50)	Case–control study (2012–2014)	Prostate cancer	Never night work	532	1	Reference
			Permanent night workers			
			Length of night shift (hours)			
			<8	11	0.32	0.16–0.64
			8–10	23	0.86	0.48–1.53
			>10	38	1.88	1.08–3.26
			Shift length (hours) for number consecutive nights			
			≤10 for <6	13	0.54	0.27–1.09
			≤10 for ≥6	18	0.58	0.32–1.07
			>10 for <6	4	0.60	0.16–2.15
			>10 for ≥6	30	2.57	1.31–5.06
			Rotating night worker			
			Length of nigh shift (hours)			
			<8	3	0.42	0.11–1.57
			8–10	69	0.79	0.56–1.12
			>10	12	1.29	0.54–3.07
			Shift length (hours) for number consecutive nights			
			≤10 for <6	13	0.72	0.49–1.06
			≤10 for ≥6	18	0.93	0.50–1.71
			>10 for <6	4	1.24	0.44–3.55
			>10 for ≥6	30	1.36	0.29–6.26
Jones et al, 2019 (88)	Cohort study (2003–2014)	Breast cancer	Average hours worked per night (hours)			
			0	1845	1	Reference
			<7	91	1.04	0.84–1.28
			≥7	103	0.96	0.78–1.17
Härmä et al 2020 (52)	Register-based case-crossover study of hospital employees in Finland (2003–2015)	Injuries	Duration of shifts (hours)			
			;≥12	440	1.23	1.06–1.42

^a^ No studies on cardio-metabolic disease were found.

**Table 4:** Studies on night shift schedules and pregnancy-related outcomes. [GW=gestational week; NA=not available; CI=confidence interval.]

Study	Study design	Outcome	Exposure	Cases	Risk	95% CI
Hammer et al, 2018 (74)	Register-based cohort study of hospital employees in Denmark (2007–2013)	Pregnancy-related hypertensive disorders (incl. pre-eclampsia)	Duration of night shifts (hours)			
			<12	214	1	Reference
			≥12	212	1.08	0.85–1.36
			Number of consecutive night shifts			
			0	132	1	Reference
			2-3	205	1.22	0.92–1.62
			≥4	89	1.41	1.01–1.98
			Number of quick returns			
			0	128	1	Reference
			2-3	203	1.12	0.87–1.45
			≥5	95	1.07	0.79–1.46
			Number of night shifts the first 20 pregnancy weeks			
			1–19	360	1	Reference
			≥20	66	1.15	0.86–1.52
Begtrup et al, 2019 (72)	Cohort study of Danish public hospital employees (2007–2013)	Miscarriage	Night shifts the previous week			
			0	1521	1	Reference
			1	167	1.00	0.85–1.18
			≥2	201	1.18	1.01–1.37
			Number of consecutive night shifts during pregnancy			
			≥2	438	1.05	0.98–1.13
			≥3	261	1.09	0.98–1.22
			≥4	93	1.16	0.97–1.38
			≥5	28	1.29	0.99–1.67
			≥6	17	1.51	1.01–2.29
			≥7	12	1.68	0.78–3.79
			Quick returns	NA	1.02	0.85-1.0
Specht et al, 2019 (89)	Register-based cohort study of hospital employees in Denmark (2007–2013)	Pre-term birth	In 2^nd^ trimester (13–22 GW)			
			Duration of night shifts (hours)			
			≤8	207	1	Reference
			>8	171	0.83	0.64–1.04
			Number of night shifts			
			1–12	320	1	Reference
			≥13	58	1.01	0.73–1.37
			Number of consecutive night shifts			
			0	106	1	Reference
			2–3	186	1.16	0.85–1.61
			≥4	86	0.98	0.68–1.42
			Number of quick returns			
			0	122	1	Reference
			1–2	68	1.19	0.85-1.64
			≥3	188	0.81	0.61–1.06
Hammer et al 2019 (73)	National register-based cohort study of Danish workers in public administration and hospitals (2007–2013)	Calling in sick within 2 days after a night shift	Day shifts	NA	1	Reference
			After a night shifts in:			
			1^st^ trimester	NA	1.28	1.19–1.37
			2^nd^ trimester	NA	1.27	1.17–1.39
			;3^rd^ trimester	NA	1.13	0.96–1.33
			Duration of night shifts (hours)			
			≤8	NA	1.20	1.12–1.30
			>8–12	NA	1.02	0.93–1.10
			>12	NA	1.55	1.43–1.69

*Intensity of night shifts*. Intensity of night shift work, which is often operationalized as number hours or night shifts per unit of time, has been suggested as important parameter in epidemiological studies (2, 42). Studies have used different measures of intensity, eg, night shift hours per week (43), lifetime mean night shifts per month (44), mean number of night shifts per week in periods with night shift work (45, 46) (table 1). Due to the large variation in the used metrics and exposure time windows, the consistency of results across studies cannot be evaluated, although generally, high intensity appears to be associated with higher risk of breast cancer (43, 45, 46), hypertension (47) and diabetes (44), but not injuries (48) (table 1).

*Number of consecutive night shifts*. Number of consecutive night shifts, ie, the number of night shifts in a row represents a specific case of intensity. Working ≥5 consecutive night shifts for ≥5 years has been associated with higher risk of breast cancer in nurses from Norway (49). Similarly, female military employees who had ≥3 consecutive night shifts for ≥6 years had higher breast cancer risk (45). Working ≥6 consecutive night shifts have been associated with a higher risk of prostate cancer, particularly in combination with long shifts, among permanent night but not rotating shift workers (50). In a meta-analysis based on eight studies mostly in industry, the risk of occupational injuries was studied on the first, second, third and fourth consecutive night shift. It was found that the risk increased with increasing number of consecutive night shifts and was highest on the fourth consecutive night shift (51). However, among hospital employees, the number of occupational injuries increased only in connection with one night shift during the past week (52). This finding can be explained by sleepiness being most profound on the first night shift as observed in both experimental and observational studies (31–38). Thus, the risk for injuries in relation to the number of consecutive night shift may depend on other shift characteristics, but at least in industry the risk has been shown to increase after three consecutive nights. Taken together, the results indicate that a maximum of three consecutive night shifts implies a lower risk of accidents and possibly cancer compared with more than three consecutive night shifts.

There are examples of night shift schedules, which involve many consecutive night shifts, eg, work on offshore oil platforms (7–14 consecutive nights) and work at remote places like Spitsbergen (21 consecutive nights), which show that circadian adaptation to night shift work appears to happen within days when it comes to subjective and objective measures of sleep (53–55) and cortisol rhythm (56). It is, however, not possible from the current literature to assess the long-term health and safety risk in these types of work settings. Thus, in special cases, eg, oilrigs and other isolated workplaces with better possibilities to adapt to daytime sleep, additional or other recommendations may apply.

*Permanent night shift work*. Permanent night shift work is characterized by having primarily or only night shifts and therefore many night shifts per week or month. It has been argued that employees with permanent night shift work adjust to day time sleep and therefore do not experience circadian disruption. However, only a very small minority of permanent night shift workers show complete adjustment of their endogenous circadian rhythm to night shift work (56, 57). We assume permanent night shift workers have health risks that are similar to workers on rotating night shift work with the addition of the potential effect of high intensity and number of consecutive night shifts. Because permanent night shift work is relatively rare, most of the existing studies that have included this work schedule are underpowered. Yet, permanent night shift work is associated with risk of breast and prostate cancer in some studies (50, 58) but not others (59, 60). The scheduling of permanent night shift work varies in duration of shifts, number of consecutive shifts and shift intervals. For this reason, the health risk associated with permanent night shift work per se cannot be evaluated without more detailed exposure information. Indeed in a study including such details, risk of prostate cancer was only higher among permanent night shift workers with long shifts and ≥6 consecutive night shifts (50).

*Shift intervals*. Shift intervals of <11 hours between two shifts (quick returns) have been associated with an increase in injuries (61, 62). Furthermore, evidence of a 5% increase in injuries for each hour less between two shifts has been shown (61). The results further indicate that the risk of injuries may be particularly increased after a quick return following a night shift (52, 61). In addition, a reduction in the number of annual quick returns reduced the risk, whereas an increase was associated with increased risk of self-reports of causing harm to one-self or patients/others (62). The available studies on shift intervals and occupational injuries indicate that risk of injury is reduced when quick returns are reduced and shift intervals are ≥11 hours (table 2).

*Direction of rotation*. In animal studies, the central circadian cycle is quicker to adjust when mimicking forward-rotation schedules, eg, D →E →N, than backward rotation, eg, N →E →D, and re-entrainment of most variables is slower for phase advance than phase delay (7). In several reviews, it has been concluded that shifting from backwards to forward rotation improves sleep (24, 39, 63), and an intervention study shows that fast forward rotation was associated with lower triglyceride and serum glucose and mean systolic blood pressure (64). Furthermore, forward rotation usually implies longer breaks (≥24 hours) when changing from one type of shift to another, whereas backward-rotation systems often imply breaks corresponding to only the duration of the shifts, and therefore may have quick returns (65). Taken together, forward-rotating schedules appear to have the most advantages. However, a few studies with prostate cancer as an outcome, which address direction of rotation and the selected outcomes, found no associations (50, 66). For this reason, the risks of forward and backward rotation in relation to cancer, cardio-metabolic diseases and injuries cannot be evaluated based on the current literature.

*Duration of night shifts*. A meta-analysis based on four studies found that risk of injury was increased with shifts lasting 10 versus 8 hours (51). A register study of hospital employees reported an increased risk for occupational injuries in shifts lasting ≥12 hours (52). A large study with pooled data from case–control studies with complete work history in five countries, found a higher risk of breast cancer in pre-menopausal (but not post-menopausal) women working night shifts lasting ≥10 hours compared with women working night shifts lasting <8 hours (46). Night shifts lasting >10 hours were associated with higher risk of prostate cancer, particularly when part of permanent night shift work and ≥6 consecutive night shifts (50). These results support that risk of injury and possibly cancer are reduced with night shifts schedules, which have shifts with a maximum duration of 9 hours (table 3).

*Pregnancy-related outcomes*. Melatonin is also produced in the placenta (67) and may play a pivotal role in proper placenta function and parturition (68, 69). Meta-analyses of several large high-quality prospective studies indicate that the risk, if any, of preterm birth and growth restriction due to night shift work is marginal (70). The evidence with respect to miscarriage has been more limited but has indicated a higher risk especially with permanent night shift work (71). Knowledge regarding risk of pregnancy-related hypertension has been limited (71). Recently, a large nationwide cohort study of health professionals using payroll data, including exact working hours during pregnancy, found a dose-dependent higher risk of miscarriage in women working ≥2 night shifts the previous week (72). In the same cohort, the risk of calling in sick within two days following night shifts was higher throughout pregnancy independent of individual factors and time-invariant confounders (73), and – among night shift workers – the risk of hypertensive disorders of pregnancy, including pre-eclampsia, grew with increasing number of consecutive night shifts, particularly among obese women (body mass index ≥30 kg/m^2^) (74). The results support that, in order to reduce risk of miscarriage, pregnant women should have no more than one night shift in a week (table 4).

### Recommendations and concluding remarks

We concluded that schedules that reduce circadian disruption may reduce cancer risk, particularly breast cancer, and schedules that optimize sleep duration and quality and reduce fatigue may reduce the occurrence of injuries. These changes in short-term physiological effects are generally achieved with fewer consecutive night shifts, sufficient shift intervals, and shorter night shift duration. Yet, sleepiness and possibly injury risk may be increased during the first night shift.

Based on the limited, existing literature, we recommend that in order to reduce the risk of injuries and possibly breast cancer, night shift schedules have (i) ≤3 consecutive night shifts, (ii) shift intervals of ≥11 hours and (iii) ≤9 hours shift duration.

In special cases, eg, oilrigs and other isolated workplaces with better possibilities to adapt to daytime sleep, other recommendations may apply.

Finally, in order to reduce risk of miscarriage, pregnant women should have no more than one night shift in a week.

The risk associated with intensity, permanent night shift work and direction of rotation could not be evaluated based on the included studies. However, high intensity appeared to be associated with higher risk of breast cancer, hypertension and diabetes, and knowledge from physiological and experimental studies are in favor of forward rotation.

Generally, major knowledge gaps were observed and the number of studies on specific night shift schedules in relation to risk of cancer, cardio-metabolic diseases and injuries is limited. Concerning cancer, the majority of studies are related to breast cancer and, to some extent, prostate cancer. There is little evidence that results from these studies are transferable to other cancer types or from one sex to the other. Nor is it known if the short-term physiological effects per se are associated with long-term health and safety risk.

There are individual differences in response to night shift work, eg, according to age and chronotype (ie, how the circadian system embeds itself into the 24-hour day). Advanced ageing is associated with earlier chronotype (75), alterations in circadian rhythmicity and sleep–wake homeostasis (76), and higher morbidity and mortality in general. Ageing is, however, not associated with higher sleepiness in general (77) or in relation to sleepiness in night shift work (78). Due to the earlier chronotype and decreased sleep efficiency, sleeping especially after the nights shifts is curtailed (79). Besides the night shifts, also quick returns are associated with increased risk for sleep problems and fatigue among those aged ≥50 (80).

The present recommendations are related to the selected night shift schedules deemed relevant for health and safety. However, there are scheduling components related to night shift work that were not covered by the expert group – such as shift start and finish times and employees’ control over night shift work. Also, the specific characteristics of night shift schedules were treated as if they were independent. However, the characteristics are highly correlated, eg, longer shift duration often implies fewer shifts and therefore more recovery time. More research is needed to establish how the different schedule characteristics interact and affect health and safety risks and if specific combinations imply a particularly higher or lower risk to health and safety. To make this possible, future etiological studies on shift work and health need to be based on precise and preferably repeated information on exposure combined with long, preferably registry-based follow-up. The use of register-based exposure information on working hours, like payroll data, is recommended if a sufficient proportion of working life is covered (81).

Although not systematic, the review of the literature aims to reduce bias in the selection of articles by employing a bibliographic search strategy and having a clear strategy for selection of papers. Several of the presented studies are based on hospital workers in the Nordic countries. Therefore, studies from other countries and sectors should be performed to take into account, eg, traditions regarding organization of night shift work, contextual factors such as working conditions and latitude-dependent exposure to sunlight over the year, work tasks, and the organization of the health care and welfare system.

Sleepiness is most profound on the first night shift. Accordingly, sleepiness is an inevitable consequence of working at night, no matter how night shifts are scheduled as long as employees change to night-time sleep during days off. Therefore, the concept of fatigue risk management, covering also sleepiness countermeasures other than optimal shift scheduling (eg, use of prior-sleep-wake data, fatigue detection technologies, and fatigue proofing strategies) has been introduced (82). Indeed, there are other ways to counteract the adverse health and safety risks of night shift work than through shift scheduling. At workplace level, fatigue risk management (82), lighting conditions (83) and self-rostering according to personal preference (84, 85) could be applied. There is some research supporting countermeasures at the individual level, such as bright light, melatonin, naps, use of stimulants, as a means to improve adaptation to night shift work (86). However, there is so far little evidence that such countermeasures reduce the long-term health consequences of night shift work (86). Lastly, other outcomes such as work–life balance and social well-being (87), productivity and patient safety may be relevant to consider when scheduling night shifts.
